# Sample size requirement in trials that use the composite endpoint major adverse cardiovascular events (MACE): new insights

**DOI:** 10.1186/s13063-022-06977-4

**Published:** 2022-12-21

**Authors:** Josep Ramon Marsal, Iratxe Urreta-Barallobre, Marimar Ubeda-Carrillo, Dimelza Osorio, Blanca Lumbreras, David Lora, Borja M. Fernández-Felix, Gerard Oristrell, Eduard Ródenas-Alesina, Lorena Herrador, Mónica Ballesteros, Javier Zamora, Jose I. Pijoan, Aida Ribera, Ignacio Ferreira-González

**Affiliations:** 1 Cardiovascular Epidemiology and Research Unit, Vall d’Hebron Institut de Recerca (VHIR), Vall d’Hebron Barcelona Hospital Campus, Pg. Vall d’Hebron, 119-129, 08035 Barcelona, Spain; 2 CIBER Epidemiology and Public Health, Madrid, Spain; 3 Biodonostia Health Research Institute, Clinical Epidemiology, San Sebastián, Spain; 4 Osakidetza Basque Health Service, Donostialdea Integrated Health Organisation, Donostia University Hospital, Clinical Epidemiology Unit, San Sebastián, Spain; 5 Osakidetza Basque Health Service, Donostialdea Integrated Health Organisation, Donostia University Hospital, Library Service, San Sebastián, Spain; 6 Health Services Research Group, Vall d’Hebron Institut de Recerca (VHIR), Vall d’Hebron Hospital Universitari, Vall d’Hebron Barcelona Hospital Campus, Passeig Vall d’Hebron 119-129, 08035 Barcelona, Spain; 7 Public Health Department, Miguel Hernandez University, Alicante, Spain; 8 Health Research Institute Hospital 12 de Octubre (imas12), Madrid, Spain; 9 Statistical Studies Department, Universidad Complutense de Madrid (UCM), Madrid, Spain; 10 Clinical Biostatistics Unit, Hospital Ramón y Cajal (IRYCIS), Madrid, Spain; 11 Cardiology Department, Vall d’Hebron University Hospital, Barcelona, Spain; 12 CIBER Cadiovascular Diseases, Madrid, Spain; 13 Institute of Metabolism and Systems Research, University of Birmingham, Birmingham, UK; 14 Clinical Epidemiology Unit, Cruces University Hospital, Barakaldo, Spain; 15 Biocruces-Bizkaia Health Research Institute, Barakaldo, Spain

**Keywords:** Composite endpoints, Sample size, Correlation, MACE

## Abstract

**Background:**

The real impact of the degree of association (DoA) between endpoint components of a composite endpoint (CE) on sample size requirement (SSR) has not been explored. We estimate the impact of the DoA between death and acute myocardial infarction (AMI) on SSR of trials using use the CE of major adverse cardiac events (MACE).

**Methods:**

A systematic review and quantitative synthesis of trials that include MACE as the primary outcome through search strategies in MEDLINE and EMBASE electronic databases. We limited to articles published in journals indexed in the first quartile of the Cardiac & Cardiovascular Systems category (Journal Citation Reports, 2015–2020). The authors were contacted to estimate the DoA between death and AMI using joint probability and correlation. We analyzed the SSR variation using the DoA estimated from RCTs.

**Results:**

Sixty-three of 134 publications that reported event rates and the therapy effect in all component endpoints were included in the quantitative synthesis. The most frequent combination was death, AMI, and revascularization (*n* = 20; 31.8%). The correlation between death and AMI, estimated from 5 trials¸ oscillated between − 0.02 and 0.31. SSR varied from 14,602 in the scenario with the strongest correlation to 12,259 in the scenario with the weakest correlation; the relative impact was 16%.

**Conclusions:**

The DoA between death and AMI is highly variable and may lead to a considerable SSR variation in a trial including MACE.

**Supplementary Information:**

The online version contains supplementary material available at 10.1186/s13063-022-06977-4.

## Background

The use of combined or composite endpoints (CEs) as primary outcomes in randomized clinical trials (RCT) is common. In the field of cardiovascular (CV) medical literature, the CE “major adverse cardiovascular event” (MACE) is widely extended [1–9]. MACE usually includes the endpoints cardiovascular or all causes of death, non-fatal acute myocardial infarction (AMI), non-fatal stroke, and, in many instances, revascularization.

The strengths and weaknesses of the use of CEs as primary outcomes have been broadly discussed [8, 9]. Their potential to reduce sample size requirement (SSR) and their ability to capture the net effect of an intervention are the most popular attributed benefits [8, 10–13]. In a recent study [14], we described how the expected occurrence rates of the individual component endpoints, the expected magnitude of the effect of the intervention on each endpoint, and the degree of association (DoA) or correlation between the component endpoints can modulate the ability of the CE to reduce the SSR [10, 14–16]. Although the expected event rate and the expected magnitude of effect on each endpoint can be estimated from prior trials, the expected DoA between endpoints of the CE is unknown. Even though this parameter is usually not incorporated in the calculation of the sample size, it may eventually dictate the ability of the CE to actually reduce the SSR [7, 9, 17–23]. Therefore, ignoring this parameter could lead to imprecise estimation of the required sample size which, in turn, can negatively impact the ethical soundness and logistical efficiency of the trial.

In the present study, we aimed to estimate the DoA between two common component endpoints of MACE (all-cause death and AMI) from a sample of clinical trials published in the CV medical literature that included MACE as their primary outcome. The ultimate purpose was to assess the impact of the DoA between these two component endpoints (all-cause death and AMI) on the SSR of a trial with a hypothetical CE combining these endpoints. A strong variability in the SSR depending on the DoA between death and AMI would justify the consideration of this parameter for a more precise calculation of sample size.

## Methods

We performed a systematic review following Preferred Reporting Items for Systematic Reviews and Meta-Analyses (PRISMA) recommendations [24]. This study was sponsored by CIBER of Epidemiology and Public Health (CIBERESP).

### Literature search

To identify studies for inclusion in this review, we developed comprehensive search strategies for MEDLINE and EMBASE electronic databases. We applied no restrictions to language or gender. The literature search was restricted to RCT using MACE as the primary outcome, published in core medical journals and indexed in the first quartile of the Cardiac & Cardiovascular Systems category in the Journal Citation Reports from 2015 to 2020.

The search strategies used, and additional core medical journals added to the criteria of selecting titles indexed in the first quartile of the Cardiac & Cardiovascular Systems category, are available in Additional file 1: Supplementary material.

### Eligibility criteria

We included RCT with (a) two-arm parallel design (*i.e., excluding crossover, factorial or more than two treatments/groups*); (b) primary outcome defined as the combination of at least 2 of the following events: cardiovascular (or all-causes) death, non-fatal AMI, and non-fatal stroke, allowing a combination with one or more other events; and (c) sample size of 500 patients or more. We excluded the manuscripts referring to interim analysis or not including the main analysis of the RCT (i.e., analysis of subgroups).

### Selection of studies and data extraction

All references’ titles and abstracts were independently screened by at least two reviewers. Second, the full article of those selected in the first screening was reviewed. This second round of screening involved two reviewers independently and was based on the application of the selection criteria. Two independent reviewers extracted the following data from each study included: population, intervention groups and outcome characteristics, intervention effects on the different outcomes, composition of the MACE, and numbers of patients with the events of interest were independently extracted by two reviewers. Finally, we contacted by email the corresponding author/s of each included RCT to request for further information and the necessary data to estimate the association between the events conforming to the MACE (see Additional file 1: Supplementary material for further details).

Any disagreement between the reviewers at all stages was solved by a third reviewer. The risk of bias was not assessed because the aim of the study was not to estimate the unbiased effect of the study interventions.

### Analytical plan

We first described the CEs of the clinical trials included in the quantitative synthesis. Then, we used the generic inverse variance method [25] to estimate the pooled event rates and the pooled risk ratios (RRs) for the CEs included in the selected trials and for those component endpoints more often included in these CEs: death (cardiovascular and all-causes death), AMI, stroke, and revascularization. This was done overall and by subgroups depending on certain RCTs characteristics: number of components of the CE, number of patients included in the trial, follow-up time, and the mean age of the population.

From the RCTs for which we could obtain the necessary data to estimate the DoA, we calculated the joint probability and Pearson’s correlation between the pair of outcomes death (all causes or cardiovascular) and AMI. We define the joint probability between the components *i* and *j* (*π*_*ij*_) as the number of patients with both components at the final assessment divided by all patients at the initial time. Pearson’s correlation between the components *i* and *j* (*ρ*_*i*, *j*_) was calculated as:

$${\rho}_{i,j}=\frac{\pi_{i,j}-{\pi}_i{\pi}_j}{\sqrt{\pi_i\left(1-{\pi}_i\right){\pi}_j\left(1-{\pi}_j\right)}}$$

where *π*_*i*_ and *π*_*j*_ are the proportion of deaths for any cause and proportion of AMI in both treatment groups (we pooled the results of both treatments since we consider unlikely different associations in each group), respectively, and *π*_*i*, *j*_ is the joint probability mentioned before.

In addition, the standardized joint probability (SJP) was calculated as *π*_*ij*_/ min(*π*_*i*_, *π*_*j*_) because the SJP has better metric properties than joint probability or correlation [26]. A value of 0 indicates no overlapping, and 1 indicates that all subjects with the less prevalent event also had the most prevalent event. We then selected the RCTs that included the outcomes death (all-causes death or CV death) and AMI as component endpoints. We estimated, from these trials, the pooled event rate in both baseline (*t* = 0) and final (*t* = 1) from the proportion of each event and the proportions of subjects with both events as follows: *π*_*t*_ = *π*_*i*, *t*_ + *π*_*j*, *t*_ − *π*_*ij*, *t*_, the pooled effect of the therapies and the SSR of a hypothetical clinical trial that included these outcomes as individual primary outcomes. SSR was calculated using the normal approximation to the binomial test [14, 27, 28] as follows:

$$n\ge {\left(\frac{Z_{{\alpha }\!\left/ \!{2}\right.}\sqrt{2\pi \left(1-\pi \right)}+{Z}_{\beta}\sqrt{\pi_{t=0}\left(1-{\pi}_{t=0}\right)+{\pi}_{t=1}\left(1-{\pi}_{t=1}\right)}}{\pi_{t=0}-{\pi}_{t=1}}\right)}^2,\pi =\frac{\pi_{t=0}+{\pi}_{t=1}}{2}$$

where ${Z}_{{\alpha }\!\left/ \!{2}\right.}$and *Z*_*β*_ are the standardized normal quantiles for error types I and II, respectively.

Finally, we estimated the pooled event rate, the pooled effect of the therapies, and the SSR in a theoretical RCT that included these endpoints as a primary CE considering two scenarios according to DoA between component outcomes: the most favorable scenario for SSR (weakest correlation) and the less favorable scenario (strongest correlation). All these calculations were further carried out considering also four hypothetical scenarios depending on the event rate of the original MACE and the relative effect of the intervention on the original MACE. Thus, scenario 1 included those studies with an event rate of the CE lower than the median event rate in the whole sample (< 10.9%) and an effect of the therapy on the CE lower than the median effect in the whole sample (RR > 0.9); scenario 2 contained those studies with an event rate of the CE lower than the median event rate in the whole sample (< 10.9%) and an effect of the therapy higher than the median effect in the whole sample (RR < 0.9); scenario 3 included those studies with an event rate > 10.9% and effect on the CE > 0.9; scenario 4 included those studies with an event rate > 10.9% and effect RR < 0.9.

## Results

### Literature search

**Fig. 1:**
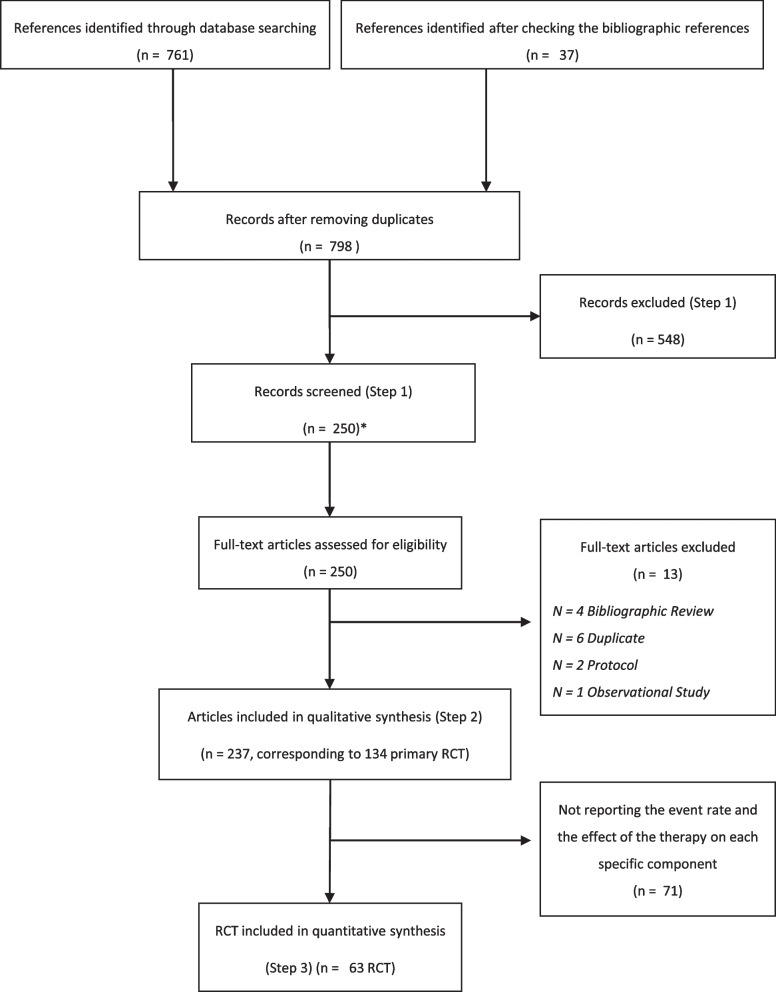
A total of 548 articles were excluded after reading the titles and abstracts, and 13 additional articles were excluded because they were duplicates or because they were not an RCT

Our literature search identified 761 articles, and 37 additional articles were detected after a manual search. A total of 548 articles were excluded after reading the titles and abstracts, and 13 additional articles were excluded because they were duplicates or because they were not an RCT (Fig. 1). The remaining 237 articles corresponded to several publications of 134 RCTs. Sixty-three out of these 134 primary publications reported both the event rate and the effect of the therapy on each specific endpoint of the MACE. These 63 were included in the quantitative synthesis. None of them reported the DoA between the endpoints of CE. The selection process is detailed in Additional file 1: Supplementary material. We sent a request for information to the corresponding authors of the 63 RCTs, and we could obtain the necessary data to estimate the DoA for only 5 RCTs.

### CE combinations

The 63 RCTs included a total of 332,199 patients (ranging from 501 to 27,564). Most trials (*n* = 38; 60.3%) included a CE with three endpoints, being AMI the most frequent (*n* = 62; 98.4%), followed by stroke (*n* = 37; 58.7%), cardiovascular death (*n* = 35; 55.6%), and revascularization (*n* = 33; 52.4%) (Table 1). The most frequent combination was death for any reason or cardiovascular death, AMI, and revascularization (*n* = 20; 31.8%). CV death and AMI were used in 32 RCTs, more than 50% of the identified trials. Death and AMI appeared in 29 RCT. In most of the analyzed trials, predetermined follow-up was longer than 1 year (*n* = 53; 84.1%).

**Table 1:** MACE’s design and characteristics of the 63 RCT included in the analysis

	Number	Percent
**RCT included**	**63**	
Type of intervention		
*Drug treatment (vs placebo)*	23	36.5
*Drug treatment (vs other drug or dose)*	5	7.9
*Procedure (type of device or invasive procedure)*	23	36.5
*Diagnostic (to guide intervention)*	9	14.1
*Other types of intervention (process of care, rehab)*	4	6.3
**No. of component outcomes**		
*3 components*	38	60.3
*4 components*	12	19.0
*5 components*	13	20.6
**No. of patients included**		
*≤ 1500*	21	33.3
*1500–5000*	22	34.9
*> 5000*	20	31.7
**Individual component outcomes**		
AMI	62	98.4
Stroke	37	58.7
CV death	35	55.6
Revascularization	33	52.4
Death for any reason	28	44.4
Angina	13	20.6
Others	9	14.3
Stent thrombosis	3	4.8
HF	3	4.8
Bleeding	4	6.3
**Most frequent MACE**		
CV death - AMI - revascularization	11	17.5
CV death - AMI - stroke	11	17.5
Death - AMI - revascularization	9	14.3
Death - AMI - stroke	6	9.5
CV death - AMI - stroke - angina	4	6.3
CV death - AMI - stroke - revascularization - angina	4	6.3
Death - AMI - revascularization - stroke	4	6.3
CV death - AMI - revascularization - angina - chest pain	1	1.6
CV death - AMI - stent thrombosis	1	1.6
CV death - AMI - stroke - angina - HF	1	1.6
CV death - AMI - stroke - bleeding	1	1.6
Death - AMI - stent thrombosis - stroke - revascularization	1	1.6
Death - AMI - angina - complicated procedure	1	1.6
Death - AMI - angina - CV admission - stroke	1	1.6
Death - AMI - HF	1	1.6
Death - AMI - revascularization - ACS	1	1.6
Death - AMI - stroke - bleeding	1	1.6
Death - AMI - stroke - bleeding - revascularization	1	1.6
Death - AMI - stroke - cardiovascular hospitalization	1	1.6
Death - low cardiac output - AMI - cardiac arrest - HF	1	1.6
Death - stroke - bleeding - vascular complication - coronary obstruction	1	1.6
**Follow-up**		
≤ 1 year	10	15.9
From 1 to 5 years	47	74.6
> 5 years	6	9.5
**Mean age over 64 years**	31	47.6

*AMI* acute myocardial Infarction, *CV death* cardiovascular death, *HF* heart failure with hospitalization, *ACS* acute coronary syndrome

Table 2 shows the pooled MACE CE event rate and the pooled effect of the different interventions in the study, expressed as risk ratio, for the overall 63 RCTs and by subgroups created according to the number of endpoints included in the CE, sample size of the trial, time of follow-up, baseline average age of the participants, and the specific endpoints included in the CE. The pooled event rate and the pooled magnitude of effect (risk ratio) for the CEs in the 63 RCT were 11.6% (95% CI 9.7–13.4) and 0.89 (95% CI 0.86–0.92), respectively. There was not any association between these parameters and the number of endpoints included in the CE. The magnitude of the effect of interventions was higher in RCTs with smaller sample sizes. However, neither time of follow-up nor the baseline mean age was associated to the effect of therapies. The largest magnitude of the effect of therapies was on the endpoint revascularization (0.75; 95% CI 0.68–0.82). Concerning the global event rate of the CEs, it was higher in those clinical trials with longer follow-up and in those with a higher baseline mean age of the population included, without differences depending on the sample size of the clinical trials. Again, the largest event rate corresponded to the endpoint revascularization (6.5%; 95% CI 7.7–8.3).

**Table 2:** MACE proportion and risk ratio depending on the different characteristics

MACE	Number	Event rate			Risk ratio		
		%	95% CI	***p***	RR	95% CI	***p***
	63	11.6	9.7–13.4%		0.89	0.86–0.92	
*No. of components*				*0.456*			*0.499*
*3 components*	*38*	*10.7*	*8.5–12.8%*		*0.91*	*0.88–0.94*	
*4 components*	*12*	*13.2*	*9.3–17.1%*		*0.87*	*0.81–0.94*	
*5 components*	*13*	*12.8*	*7.4–18.1%*		*0.87*	*0.81–0.94*	
*No. of patients included*				*0.428*			*0.002*
*≤ 1500*	*21*	*13.0*	*9.2–16.8%*		*0.77*	*0.71–0.85*	
*1500–5000*	*22*	*11.9*	*9.5–14.3%*		*0.91*	*0.89–0.94*	
*> 5000*	*20*	*9.9*	*6.8–13.1%*		*0.91*	*0.86–0.95*	
*Follow-up time*				*0.002*			*0.650*
*≤ 1 year*	*10*	*11.9*	*6.7–17.1%*		*0.85*	*0.78–0.94*	
*1 to 5 years*	*47*	*10.2*	*8.4–11.9%*		*0.89*	*0.86–0.93*	
*> 5 years*	*6*	*22.0*	*15.7–28.3%*		*0.89*	*0.83–0.98*	
*Age*				*0.039*			*0.907*
*≤ 64 years*	*32*	*9.8*	*7.3–12.4%*		*0.89*	*0.85–0.92*	
*> 64 years*	*31*	*13.6*	*11.1–16.2%*		*0.89*	*0.85–0.93*	
*Individual outcome*							
*CV death*		3.5	*2.7–4.3%*		*0.9*	*0.87–0.94*	
*All-causes death*		3.8	*2.6–5%*		*0.88*	*0.82–0.94*	
*Acute myocardial infarction*		4.3	*3.4–5.1%*		*0.87*	*0.83–0.91*	
*Stroke*		1.7	*1.4–2.1%*		*0.84*	*0.79–0.88*	
*Revascularization*		6.5	*4.7–8.3%*		*0.75*	*0.68–0.82*	

### Impact of correlation between death and MI on SSR

**Fig. 2:**
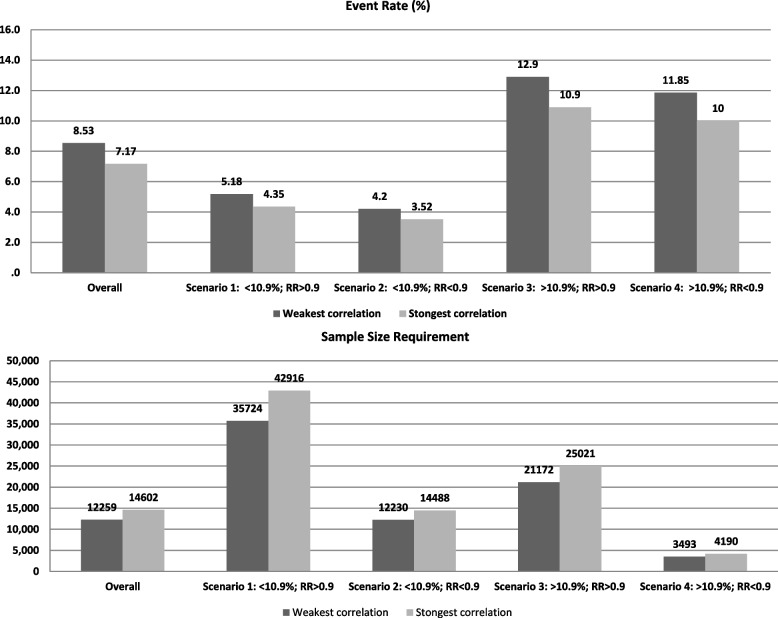
Impact of the correlation between death and acute myocardial infarction on the event rate and sample size requirement of a theoretical trial that includes these outcomes as a composite endpoint

Table 3 shows the DoA between death and AMI estimated from 5 studies and expressed as the joint probability of the distributions of both endpoints and the correlation, which ranged between − 0.02 and 0.31. Figure 2 shows the impact of the correlation between death and acute myocardial infarction on the event rate and on the SSR of a theoretical trial that includes these outcomes in a composite endpoint. All calculations were performed with data from the overall 63 clinical trials (left columns in the figure) and classifying these trials in four scenarios according to the pooled magnitude of the effect of the intervention on the original CE (RR < 0.9 and RR > 0.9) and the pooled CE event rate (< 10.9% and > 10.9%).

**Table 3:** Degree of association between death and acute myocardial infarction, expressed by joint probability and correlation, in the 5 RCT with complete information

Study	Number	Death (***n***, (%))	AMI (***n***, (%))	Death and AMI (***n***)	Joint probability (%)	Correlation (95% CI)
ACCELERATE [29]^a^	12,092	309 (2.6)	517 (4.3)	74	0.61	0.157 (0.14*–*0.17)
GIK [30]	930	20 (2.2)	2 (0.2)	2	0.22	0.31 (0.25*–*0.37)
PROMISE [31]	10,003	149 (1.5)	70 (0.7)	3	0.03	0.02 (0*–*0.04)
Yoga-Ca [32]	3,959	144 (3.6)	28 (0.7)	0	0.00	− 0.02 (− 0.05*–*0.01)
MR.INFORM [33]	918	6 (0.7)	19 (2.1)	1	0.11	0.08 (0.02*–*0.14)

*AMI* Acute myocardial infarction

^a^Cardiovascular death

Considering the full set of RCT, the impact of correlation on the event rate was a variation of 19% from the less favorable scenario (strongest correlation, event rate 7.17%) to the most favorable scenario (weakest correlation, event rate 8.53%). This led to a SSR variation of 16% from the less favorable scenario (strongest correlation, SSR 14,602 patients) to the most favorable scenario (weakest correlation, SSR 12,259 patients), which means a difference of 2343 patients in absolute numbers. Although the relative impact of the DoA across the four scenarios was similar, in absolute values, SSR may vary up to 7192 patients, depending upon the specific combination of absolute risk of the event in the study follow-up and the magnitude of the relative effect of the intervention.

## Discussion

In this systematic review of 63 randomized clinical trials in the CV field published in first-quartile journals, the CE most often used (*n* = 20 trials) included the endpoints death (all-cause death or CV death), AMI, and revascularization. In the pooled analysis of these RCTs, the pooled effect of therapies and the pooled event rate were higher in the component endpoint revascularization, which is clinically the less important outcome. The DoA between the endpoints death and AMI, which could be estimated from 5 RCTs, was highly variable. This variability led to a SSR variation of a hypothetical CE including these two outcomes from 2343 patients (16%). The relative impact of the DoA on the variation of SSR was similar in different settings regardless of the event rate and magnitude of the effect of the intervention, but, in absolute terms, SSR variation may reach a maximum of 7192 patients.

In previous publications, we and others already showed that both the CE event rate and the effect of the therapy on the CE are usually driven by the less important endpoint [6, 10, 14, 15, 34]. In this systematic review of a sample of CV trials that used the CE MACE as the primary endpoint, we analyzed the pooled event rate and the pooled effect of the therapies on the most common endpoints, such as death, AMI, stroke, and revascularization. Again, the event rate was higher, and the magnitude of the effect of therapy was larger on the endpoint revascularization, which is the less important and can be considered a clinician-driven outcome. The gradients in both importance to patients and treatment effects across component endpoints may complicate the interpretation of trial results, as was previously pointed out [7]. Regardless of the potential problems of interpretation due to a gradient between endpoints, since one of the main uses of the CE is to reduce SSR, in this work, we explore how the interrelationship between the most popular components included in the CE MACE (i.e., death and AMI) may impact SSR.

As we illustrated in a simulation study, the interrelationship between endpoints included in a CE can help to define the SSR in some situations, at least in the cardiovascular field. Specifically, we showed that in 25 of 66 simulated scenarios the DoA between two CV endpoints influenced the decision of using a CE instead of a single endpoint [14, 16]. We also showed that the influence of the DoA in SSR decreases when the effect of the therapy on the relevant endpoint increases.

Although it is true that the smaller the DoA between endpoints the lower the SSR of the corresponding CE, the impact of the DoA on SSR is also importantly influenced by the CE event rate and by the magnitude of the effect of the therapy on endpoints, as we previously have shown [14]. Thus, from a statistical perspective, a small DoA between two specific endpoints should not lead to the selection of these endpoints for a CE to decrease the SSR. Rather, the expected effect of the therapy on the endpoints, the expected event rate, and, importantly, the specific type of endpoints should carefully be considered during the sample size calculation. In this sense, our findings could be useful for sample size considerations in future cardiovascular trials using the CE MACE. The present work shows that the variability of the DoA between death and AMI endpoints, estimated from real CV trials, is generally high. Specifically, the required sample size for a trial with a CE combining this couple of endpoints might vary as much as 17% depending on the DoA between them, which supposes a considerable uncertainty.

We also analyzed the impact of the DoA on the SSR across four scenarios defined by the CE event rate and by the effect of the therapy on the CE (Fig. 2). This impact, in relative terms, was similar across the four scenarios, probably because the ranges of the event rates and the magnitudes of the effect of the experimental interventions among the trials included were narrow, and these parameters are closely related with the impact of the DoA on the SSR, as we previously showed [14].

Nowadays, it is impossible to know in advance the DoA between the component endpoints of a CE. For this reason, our initial approach has been to estimate the range of variation of the DoA between the most popular endpoints included in the CE MACE, as a first step to understand the potential of this parameter to drive the SSR. In any case, the DoA between CV endpoints may likely be influenced by other intrinsic characteristics of the population, such as the baseline cardiovascular risk. For instance, the probability of death in patients who had an AMI is likely to be higher in patients with diabetes, hypertension, and renal impairment than in patients without these conditions, which can lead to a different DoA estimation between these endpoints. Similarly, clinician-driven outcomes, such as revascularization, could be influenced by regional practices, which can also lead to different DoA between revascularization and other CV outcomes depending on the region. Therefore, the next step in future research should be to explore in depth, in large databases from RCT, or in large observational studies or registries, not only the DoA between other CV endpoints but which factors, if any, determine a stronger or weaker DoA between different CV endpoints. This will permit us to anticipate the expected DoA more precisely, and thus, it will lead to a more informed and precise sample size estimation in future studies. In the meantime, given the important variation in correlation estimates between death and AMI, ranging from − 0.02 to 0.31, there are several possibilities for sample size estimation when using these outcomes. One could conservatively use the worst case, one could use some average correlation, or one could estimate the correlation in a blinded interim analysis and recalculate the sample size. Although our intention is not to make a formal recommendation regarding how to calculate SSR based on DoA, we think that the latter strategy would likely allow a more precise correction in the interim analysis regarding SSR.

## Limitations

Unfortunately, we could obtain the data necessary to estimate the DoA between death and AMI only from 5 RCTs. Therefore, we cannot conclude that our DoA estimations may be representative of the full spectrum of CV trials. However, these five trials may represent, to a certain extent, a disparate trial sample (i.e., different sample sizes, event rates, magnitude of effect). We also restricted ourselves to the study of CV CE, specifically MACE, a commonly used endpoint. Relationships between component endpoints making up CE in other fields (e.g., infectious and neurological diseases) may be different, as well as their potential impact on the required sample size for trials in those clinical areas.

Another limitation of our study is that we restricted our approximation to the differences in proportions, while most clinical trials in the cardiovascular area use survival analysis to estimate hazard rations. In addition, the fact that we do not consider censoring could yield biased estimates. Although we attempt to study how the association between the most usual outcomes in the cardiovascular field varies and how this variation may impact the SSR estimations, future studies should focus on survival data to quantify DoAs between two components of a MACE, which is probably a more realistic approach.

## Conclusions

The use of CE in clinical trials is a challenge for the interpretation of their results and for the estimation of the anticipated SSR. The most common CE used in CV trials combines the outcomes death, AMI, stroke, and revascularization. In this CE, the higher event rate and the higher effect of therapies usually falls on the outcome revascularization, which is the less important. The DoA between components of the CE MACE may influence the SSR. Specifically, the DoA between the endpoints death and MI is highly variable and can lead to a sizeable SSR variation of a trial with a theoretical CE including these outcomes. Therefore, the DoA among the components of the most frequent CEs used in clinical trials should be explored in depth in future studies. We recommend that future trialists incorporate some measures of the degree of association between components in their publications. Also, initiatives for enhancing transparency in publications of health research, such as EQUATOR, could also help tackle the lack of recommendations on what information to display and how to display it when CEs are used as the main results of a clinical study. This would permit the incorporation of this parameter for a refined calculation of the sample size in future clinical trials.

## Supplementary Information

**Additional file 1: Supplementary material.**

## Data Availability

All data and materials are available from the corresponding author upon request.

## Abbreviations

AMI: Acute myocardial infarction
CEs: Composite endpoints
CV: Cardiovascular
DoA: Degree of association
MACE: Major adverse cardiac events
PRISMA: Preferred Reporting Items for Systematic Reviews and Meta-Analyses
RCT: Randomized clinical trials
RR: Risk ratio
SSR: Sample size requirement
