# Supplementary material for: Sample size requirement in trials that use the composite endpoint major adverse cardiovascular events (MACE): new insights
Source: Trials. 2022 Dec 21;23:1037. doi: 10.1186/s13063-022-06977-4 (PMC9769015; doi:10.1186/s13063-022-06977-4)
Supplement: Supplementary file 1 — Additional file 1: Supplementary material. [file 13063_2022_6977_MOESM1_ESM.docx]

Contenido

[SEARCH STRATEGIES: MEDLINE, EMBASE 1](#_Toc87257101)

[DATA EXTRACTION 6](#_Toc87257102)

[Table 1. Agreement and concordance between reviewer pairs 8](#_Toc87257103)

[Table 2. List of included Randomized Clinical Trials 11](#_Toc87257104)

[Table 3. Degree of association between each pair of events quantified by the Joint probability and the Correlation 13](#_Toc87257105)

[Bibliography 15](#_Toc87257106)

## SEARCH STRATEGIES: MEDLINE, EMBASE

**Database: Ovid MEDLINE(R) ALL / PubMed(R) <1946 to Present>**

**Search Strategy:**

1 mace.tw.

2 major adverse cardiac event$.mp.

3 major cardiac adverse event$.mp. [mp=title, abstract, original title, name of substance word, subject heading word, floating sub-heading word, keyword heading word, organism supplementary concept word, protocol supplementary concept word, rare disease supplementary concept word, unique identifier, synonyms]

4 major adverse cardiovascular event$.mp. [mp=title, abstract, original title, name of substance word, subject heading word, floating sub-heading word, keyword heading word, organism supplementary concept word, protocol supplementary concept word, rare disease supplementary concept word, unique identifier, synonyms]

5 1 or 2 or 3 or 4

6 exp cerebrovascular accident/

7 (Cerebrovascular adj1 (accident$ or arrest or failure or injury or insufficiency or insult)).mp. [mp=title, abstract, original title, name of substance word, subject heading word, floating sub-heading word, keyword heading word, organism supplementary concept word, protocol supplementary concept word, rare disease supplementary concept word, unique identifier, synonyms]

8 (stroke$ or apoplex? or brain vascular accident$ or cva).mp. [mp=title, abstract, original title, name of substance word, subject heading word, floating sub-heading word, keyword heading word, organism supplementary concept word, protocol supplementary concept word, rare disease supplementary concept word, unique identifier, synonyms]

9 (cerebral adj1 (stroke$ or insult)).mp. [mp=title, abstract, original title, name of substance word, subject heading word, floating sub-heading word, keyword heading word, organism supplementary concept word, protocol supplementary concept word, rare disease supplementary concept word, unique identifier, synonyms]

10 (vascular adj2 accident$ adj2 brain).mp. [mp=title, abstract, original title, name of substance word, subject heading word, floating sub-heading word, keyword heading word, organism supplementary concept word, protocol supplementary concept word, rare disease supplementary concept word, unique identifier, synonyms]

11 (brain adj1 (accident or attack or blood flow disturbance or insultus or ischaemic attack or ischemic attack or vascular accident)).mp. [mp=title, abstract, original title, name of substance word, subject heading word, floating sub-heading word, keyword heading word, organism supplementary concept word, protocol supplementary concept word, rare disease supplementary concept word, unique identifier, synonyms]

12 or/6-11

13 exp heart infarction/

14 ((cardiovascular stroke$ or heart) adj1 (attack$ or infarct$) adj1 myocardi$ infarct$).mp. [mp=title, abstract, original title, name of substance word, subject heading word, floating sub-heading word, keyword heading word, organism supplementary concept word, protocol supplementary concept word, rare disease supplementary concept word, unique identifier, synonyms]

15 13 or 14

16 mortality.mp. or mortality/

17 death/ or cadiovascular death.mp. [mp=title, abstract, original title, name of substance word, subject heading word, floating sub-heading word, keyword heading word, organism supplementary concept word, protocol supplementary concept word, rare disease supplementary concept word, unique identifier, synonyms]

18 16 or 17

19 12 or 15 or 18

20 (composite adj1 endpoint$).mp. [mp=title, abstract, original title, name of substance word, subject heading word, floating sub-heading word, keyword heading word, organism supplementary concept word, protocol supplementary concept word, rare disease supplementary concept word, unique identifier, synonyms] (3518)

21 19 and 20

22 5 or 21

23 limit 22 to yr="2015 -Current"

24 limit 23 to "therapy (maximizes specificity)"

**Database: Embase <1974 to 2021 Week 10>**

**Search Strategy:**

1 mace.tw.

2 major adverse cardiac event$.mp.

3 major cardiac adverse event$.mp. [mp=title, abstract, heading word, drug trade name, original title, device manufacturer, drug manufacturer, device trade name, keyword, floating subheading word, candidate term word]

4 major adverse cardiovascular event$.mp. [mp=title, abstract, heading word, drug trade name, original title, device manufacturer, drug manufacturer, device trade name, keyword, floating subheading word, candidate term word]

5 1 or 2 or 3 or 4

6 exp cerebrovascular accident/

7 (Cerebrovascular adj1 (accident$ or arrest or failure or injury or insufficiency or insult)).mp. [mp=title, abstract, heading word, drug trade name, original title, device manufacturer, drug manufacturer, device trade name, keyword, floating subheading word, candidate term word]

8 (stroke$ or apoplex? or brain vascular accident$ or cva).mp. [mp=title, abstract, heading word, drug trade name, original title, device manufacturer, drug manufacturer, device trade name, keyword, floating subheading word, candidate term word]

9 (cerebral adj1 (stroke$ or insult)).mp. [mp=title, abstract, heading word, drug trade name, original title, device manufacturer, drug manufacturer, device trade name, keyword, floating subheading word, candidate term word]

10 (vascular adj2 accident$ adj2 brain).mp. [mp=title, abstract, heading word, drug trade name, original title, device manufacturer, drug manufacturer, device trade name, keyword, floating subheading word, candidate term word]

11 (brain adj1 (accident or attack or blood flow disturbance or insultus or ischaemic attack or ischemic attack or vascular accident)).mp. [mp=title, abstract, heading word, drug trade name, original title, device manufacturer, drug manufacturer, device trade name, keyword, floating subheading word, candidate term word]

12 or/6-11

13 exp heart infarction/

14 ((cardiovascular stroke$ or heart) adj1 (attack$ or infarct$) adj1 myocardi$ infarct$).mp. [mp=title, abstract, heading word, drug trade name, original title, device manufacturer, drug manufacturer, device trade name, keyword, floating subheading word, candidate term word]

15 13 or 14

16 mortality.mp. or mortality/

17 death/ or cadiovascular death.mp. [mp=title, abstract, heading word, drug trade name, original title, device manufacturer, drug manufacturer, device trade name, keyword, floating subheading word, candidate term word]

18 16 or 17

19 12 or 15 or 18

20 (composite adj1 endpoint$).mp. [mp=title, abstract, heading word, drug trade name, original title, device manufacturer, drug manufacturer, device trade name, keyword, floating subheading word, candidate term word]

21 19 and 20

22 5 or 21

23 limit 22 to yr="2015 -Current"

24 limit 23 to "therapy (maximizes specificity)"

**Additional Core Medical Journals added to the criteria of selecting titles indexed in the first quartile of the Cardiac & Cardiovascular Systems**

Annals of Internal Medicine

BMJ - British Medicine Journal

JAMA - Journal of American Medical Association

New England Journal of Medicine

LANCET

## DATA EXTRACTION

**STEP 1: Title and abstract reading**

The investigators were randomly assigned into pairs. Titles and abstracts were classified into: a) potential inclusion, b) Duplicate, c) Other language (neither English nor Spanish), d) Not meeting the Inclusion/Exclusion criteria or d) Systematic Review or Meta-analysis. When it did not exist agreement between both labels and one of them was ‘*potentially includible*’, JRM and AR decided about the inclusion of the manuscript to the following step. Figure 1 depicts the flow-chart of the step 1.

Figure 1. References selected after abstract reading (step 1).

**C**

**B**

**A**

**761**

**213**

34 (22.5%)

54 (35.3%)

44 (28.8%)

28 (18.3%)

53 (35.1%)

Not Agreement*: n=36 (23.5%)

*Potential Inclusion:* ***6 (3.4%)***

Pair 5 (N = 151)

*Agreement: 101 (66.9%)*

*Potential Inclusion:* ***21 (13.9%)***

Pair 4 (N = 153)

*Agreement: 113 (73.9%)*

*Potential Inclusion****: 48 (31.4%)***

Pair 1 (N = 151)

*Agreement: 117 (77.5%)*

*Potential Inclusion:* ***49 (32.5%)***

Pair 2 (N = 153)

*Agreement: 102 (66.7%)*

*Potential Inclusion:* ***14 (9.2%)***

Pair 3 (N = 153)

*Agreement: 117 (76.5%)*

*Potential Inclusion:* ***33 (21.6%)***

Not Agreement*: n=29 (19.0%)

*Potential Inclusion:* ***11 (7.2%)***

Not Agreement*: n=45 (29.8%)

*Potential Inclusion:* ***13 (8.6%)***

Not Agreement*: n=40 (26.1%)

*Potential Inclusion:* ***14 (9.2%)***

Not Agreement*: n=30 (19.9%)

*Potential Inclusion:* ***4 (2.6%)***

*Panel A: Number of references assigned. Number and proportion of agreement. Number and proportion of potential inclusion reference.*

*Panel B: Not Agreement*: Number and proportion of references without agreement but one of them received the label of ‘potential inclusion’. Number and Proportion of potential inclusion after JRM and AIR read.*

*Panel C: Number and proportion of references finally included at the next step for each pair of readers.*

**STEP 2: Full-text Reading.**

We included 37 new references not detected from the literature research to the 213 initial included. These 37 references were detected from the meta-analysis and reviews discarded initially. From these 250 references, we excluded 13 for the following reasons: 4 bibliographic reviews, 6 duplicated, 2 were protocols and 1 observational study. Among the 237 remaining references, 57 oral communication or posters were excluded and 1 reference was also excluded because it was not possible to find the full-text. Once all the full-texts were obtained we classified them in 134 unique primary studies RCT (Supplementary Material II - Table 3).

**STEP 3: Data extraction from RCT**

The 134 RCT were randomly distributed between 8 pair of specifically-trained investigators. These investigators read the full-text, taking special attention at the inclusion/exclusion criteria, checked the possible bias and if the RCT was includible they extracted the data.

63 RCT out of 134 RCT, were finally included. 39.

## Table 1. Agreement and concordance between reviewer pairs

|  |  |  | **Reviwer1** | **Reviwer2** |
| --- | --- | --- | --- | --- |
| **Pair 1** | Missing Data | *n(%)* | 0 (0%) | 0 (0%) |
|  |  | *N* | 16 | 16 |
|  | Included | *n(%)* | 0 (0%) | 0 (0%) |
|  | Included with lack of information |  | 9 (56.2%) | 4 (25%) |
|  | Rejected |  | 7 (43.8%) | 12 (75%) |
|  | Agreement | *n / %(IC95%)* | 11 | 68.8% [46%-91.5%] |
|  | Concordance | *Kappa / IC95%* | 0.412 | 0.066-0.757 |
| **Pair 2** | Missing Data | *n(%)* | 0 (0%) | 0 (0%) |
|  |  | *N* | 17 | 17 |
|  | Included | *n(%)* | 0 (0%) | 0 (0%) |
|  | Included with lack of information |  | 9 (52.9%) | 6 (35.3%) |
|  | Rejected |  | 8 (47.1%) | 11 (64.7%) |
|  | Agreement | *n / %(IC95%)* | 14 | 82.4% [64.2%-100.5%] |
|  | Concordance | *Kappa / IC95%* | 0.653 | 0.319-0.987 |
| **Pair 3** | Missing Data | *n(%)* | 0 (0%) | 0 (0%) |
|  |  | *N* | 17 | 17 |
|  | Included | *n(%)* | 0 (0%) | 6 (35.3%) |
|  | Included with lack of information |  | 8 (47.1%) | 3 (17.6%) |
|  | Rejected |  | 9 (52.9%) | 8 (47.1%) |
|  | Agreement | *n / %(IC95%)* | 8 | 47.1% [23.3%-70.8%] |
|  | Concordance | *Kappa / IC95%* | 0.207 | -0.05-0.464 |
| **Pair 4** | Missing Data | *n(%)* | 0 (0%) | 0 (0%) |
|  |  | *N* | 16 | 16 |
|  | Included | *n(%)* | 1 (6.2%) | 0 (0%) |
|  | Included with lack of information |  | 0 (0%) | 7 (43.8%) |
|  | Rejected |  | 15 (93.8%) | 9 (56.2%) |
|  | Agreement | *n / %(IC95%)* | 9 | 56.2% [31.9%-80.6%] |
|  | Concordance | *Kappa / IC95%* | 0.074 | -0.054-0.203 |
| **Pair 5** | Missing Data | *n(%)* | 1 (5.9%) | 1 (5.9%) |
|  |  | *N* | 16 | 16 |
|  | Included | *n(%)* | 0 (0%) | 0 (0%) |
|  | Included with lack of information |  | 8 (50%) | 7 (43.8%) |
|  | Rejected |  | 8 (50%) | 9 (56.2%) |
|  | Agreement | *n / %(IC95%)* | 15 | 93.8% [81.9%-105.6%] |
|  | Concordance | *Kappa / IC95%* | 0.875 | 0.64-1 |
| **Pair 6** | Missing Data | *n(%)* | 0 (0%) | 0 (0%) |
|  |  | *N* | 16 | 16 |
|  | Included | *n(%)* | 1 (6.2%) | 0 (0%) |
|  | Included with lack of information |  | 9 (56.2%) | 9 (56.2%) |
|  | Rejected |  | 6 (37.5%) | 7 (43.8%) |
|  | Agreement | *n / %(IC95%)* | 14 | 87.5% [71.3%-103.7%] |
|  | Concordance | *Kappa / IC95%* | 0.759 | 0.463-1 |
| **Pair 7** | Missing Data | *n(%)* | 0 (0%) | 0 (0%) |
|  |  | *N* | 16 | 16 |
|  | Included | *n(%)* | 0 (0%) | 0 (0%) |
|  | Included with lack of information |  | 8 (50%) | 8 (50%) |
|  | Rejected |  | 8 (50%) | 8 (50%) |
|  | Agreement | *n / %(IC95%)* | 14 | 87.5% [71.3%-103.7%] |
|  | Concordance | *Kappa / IC95%* | 0.75 | 0.426-1 |
| **Pair 8** | Missing Data | *n(%)* | 0 (0%) | 0 (0%) |
|  |  | *N* | 16 | 16 |
|  | Included | *n(%)* | 0 (0%) | 1 (6.2%) |
|  | Included with lack of information |  | 8 (50%) | 6 (37.5%) |
|  | Rejected |  | 8 (50%) | 9 (56.2%) |
|  | Agreement | *n / %(IC95%)* | 10 | 62.5% [38.8%-86.2%] |
|  | Concordance | *Kappa / IC95%* | 0.294 | -0.123-0.712 |
| **ALL** | Missing Data | *n(%)* | 1 (0.8%) | 1 (0.8%) |
|  |  | *N* | 130 | 130 |
|  | Included | *n(%)* | 2 (1.5%) | 7 (5.4%) |
|  | Included with lack of information |  | 59 (45.4%) | 50 (38.5%) |
|  | Rejected |  | 69 (53.1%) | 73 (56.2%) |
|  | Agreement | *n / %(IC95%)* | 95 | 73.1% [65.5%-80.7%] |
|  | Concordance | *Kappa / IC95%* | 0.489 | 0.353-0.624 |

**STEP 4: Mailing**

The 63 corresponding authors were invited to participate in the study. After two e-mails, 38 did not respond, 7 declined the invitation and 5 participated. Supplementary Material II- Table 5 contains the DoA reported by the 5 correspondence authors.

*Mail #1*

*Dear Dr. <corresponding author>*

*My name is Josep Ramon Marsal, Investigator at the Cardiovascular Research Unit of the Vall d’Hebron University Hospital in Barcelona, Spain.*

*We have read with interest your article <TITLE> on <BIBLIOGRAPHIC REFERENCE>*

*Your publication will be included in a systematic review and meta-analysis to analyse the magnitude of the association between cardiovascular endpoints used in a MACE. Specifically, this systematic review is part of a project to improve Bin-CE, a free tool to estimate sample size requirements for clinical trials (available at*[*https://uesca-apps.shinyapps.io/bincep/*](https://uesca-apps.shinyapps.io/bincep/)*). We will build a repository with the associations of the most common pairs of events combined in a MACE (i.e. AMI, stroke, death) to help trialists in sample size calculations. Bin-CE is being funded by CIBER of Epidemiology and Public Health, a center of excellence in epidemiological investigation from the Spanish Government.*

***We kindly ask you to provide this information if possible.***

*Would you agree to participate, please find attached an excel file with instructions where you can fill in the information we require (shadowed cells). Do not hesitate to contact me if you need more information about the project or if you need support to complete the Excel file.*

*Your collaboration will be recognized in all products of this work (i.e. articles, web-tool).*

*We thank you in advance for your time and effort.*

*Yours sincerely,*

*Ignacio Ferreira-González MD, PhD, FESC and Josep Ramon Marsal PhD*

*Mail #2*

*Dear <corresponding author>*

*Some days ago we send you an e-mail inviting you and your team to participate in a meta-analysis. Specifically, we contacted you because we read with interest the results of the* ***<TRIAL NAME>****. The objective of our study is to assess the association between components included in a MACE and their determinants. Since the* ***<TRIAL NAME>****used a MACE as a primary endpoint, it is a candidate to be included in this meta-analysis.*

*Should you be interested in participating, we kindly ask you to fulfill the Excel file attached to this e-mail. It will only take 10-15 minutes if the requested data are available.*

*We would really appreciate your participation in this project and* ***we invite you to form part of the authorship*** *of the products of the research (publications and Bin-CE calculator).*

*If you need further information you can contact us using this e-mail address or by phone at +34 636 57 57 96 or +34 93 274 61 77.*

*I look forward to hearing from you soon.*

*Sincerely,*

*Ignacio Ferreira-González MD, PhD, FESC and Josep Ramon Marsal PhD*

## Table 2. List of included Randomized Clinical Trials

| **Id** | **RCT** | **Corresponding Author** | **Bibliographic reference** |
| --- | --- | --- | --- |
| 1 | ABSORB II(1) | Patrick W. Serruys | *EuroIntervention. 2016 Oct 1;12(9):1102–7* |
| 2 | ACCELERATE(2) | Dr Lincoff | *The New England Journal of Medicine 2017; 376: 1933-42* |
| 5 | AIDA STEMI(3) | Dr Holger Thiele | *Lancet. 2012 Mar 10;379(9819):923-931* |
| 9 | ARTEMIS(4) | Tracy Y. Wang | *JAMA. 2019 Jan 1;321(1):44-55* |
| 10 | ASCENT-COPD(5) | Robert A. Wise,MD | *JAMA. 2019;321(17):1693-1701.* |
| 11 | ATLANTIC(6) | Dr. Montalescot | *N Engl J Med 2014;371:1016-27.* |
| 13 | BASE ACS(7) | Karjalainen PP | *International Journal of Cardiology 222 (2016) 275–280* |
| 15 | BEST(8) | Dr. Seung-Jung Park | *N Engl J Med. 2015 Mar 26;372(13):1204-12* |
| 16 | BETonMACE(9) | Kausik K Ray | *JAMA. 2020 Apr 28;323(16):1565-1573* |
| 18 | BIOSTEMI(10) | Prof Stephan Windecker | *Lancet 2019; 394: 1243-1253* |
| 20 | BRAVO-3(11) | Dr. George D. Dangas | *J Am Coll Cardiol. 2015 Dec 29;66(25):2860-2868* |
| 23 | CARMELINA(12) | Marx N, McGuire D | *European Heart Journal. 2019;40(Supplement_1)* |
| 24 | CATCH(13) | Dr. Jesper J. Linde | *JACC Cardiovasc Imaging. 2015 Dec;8(12):1404-1413* |
| 31 | CONSERVE(14) | Dr. James K. Min | *J Am Coll Cardiol Img 2019;12:1303–12* |
| 33 | Compare-Acute(15) | Pieter C. Smits | *CARDIOVASCULAR INTERVENTIONS VOL 2017; 10(12):1215-21* |
| 37 | DECISION-CTO(16) | Seung-Jung Park | *circulation. 2019 Apr 2;139(14):1674-1683* |
| 38 | DECLARE-TIMI 58(17) | Wiviott S, Raz I | *New England Journal of Medicine. 2019;380(4):347-357* |
| 39 | DEFINE-FLAIR(18) | Dr Davies | *The New England Journal of Medicine2017; 376: 1824-34* |
| 40 | DESSOLVE-III(19) | Patrick W Serruys. | *The lancet 2018; 391: 431-440* |
| 43 | ELIXA(20) | Dr. Pfeffer | *N Engl J Med. 2015 Dec 3;373(23):2247-57* |
| 46 | EMPA-REG OUTCOME(21) | Christoph Wanner | *N Engl J Med. 2016 Jul 28;375(4):323-34.* |
| 48 | EUCLID(22) | Dr. Patel | *N Engl J Med. 2017 Jan 5;376(1):32-40* |
| 52 | EXSCEL(23) | Dr. Holman | *New England Journal of Medicine. 2017;377(13):1228-1239* |
| 53 | FAME(24) | Nico H J Pills | *Lancet. 2015 Nov 7;386(10006):1853-60* |
| 55 | FOURIER(25) | Marc S. Sabatine | *The New England Journal of medicine 2017; 376: 1713-22* |
| 57 | HARMONY(26) | Professor John J V McMurray | *Lancet 2018; 392: 1519–29* |
| 60 | iFR Swedeheart(27) | Dr Göetberg | *The New England Journal of medicine 2017; 376: 1813-23* |
| 61 | IMPROVE-IT(28) | Dr. Cannon | *N Engl J Med. 2015 Jun 18;372(25):2387-97* |
| 63 | ITALIC(29) | Prof. Martine Gilard | *JACC Cardiovasc Interv. 2017 Jun 26;10(12):1202-1210* |
| 64 | IVUS-XPL(30) | Myeong-Ki Hong, MD, PhD | *JAMA. 2015;314(20):2155-2163* |
| 65 | J-DESsERT(31) | M. Nakamura | *International Journal of Cardiology 208 (2016) 4–12* |
| 67 | LEADER(32) | John B. Buse | *New England Journal of Medicine. 2016;375(4):311-22* |
| 70 | MATRIX(33) | Dr.Valgimigli | *N Engl J Med. 2015; 373(11): 997 - 1009* |
| 72 | MK-3102(34) | Ira Gantz | *Cardiovasc Diabetol. 2017 Sep 11;16(1):112* |
| 73 | MR INFORM(35) | Dr. Nagel | *N Engl J Med. 2019 Dec 5;381(23):2277-2278* |
| 74 | NOBLE(36) | Evald Hoj Christiansen | *Lancet. 2020 Jan 18;395(10219):191-199* |
| 76 | ODYSSEY OUTCOMES(37) | Dr. Schwartz | *N Engl J Med. 2018 Nov 29;379(22):2097-2107* |
| 77 | ONYX ONE(38) | Dr. Windecker | *N Engl J Med. 2020 Mar 26;382(13):1208-1218* |
| 82 | PHARMCLO(39) | Diego Ardissino | *J Am Coll Cardiol. 2018 May 1;71(17):1869-1877* |
| 84 | PIONEER 6(40) | Mansoor Husain | *N Engl J Med 2019;381:841-51* |
| 86 | PRECOMBAT(41) | Dr S-J Park | *N Engl J Med 2011; 364:1718-27* |
| 92 | REDUCE-IT(42) | Dr. Bhatt | *N Engl J Med 2019;380:11-22* |
| 93 | REGROUP(43) | Marco A. Zenati | *N Engl J Med 2019;380:132-41* |
| 94 | RESOLUTE(44) | Patrick W. Serruys, MD, PhD | *Circ Cardiovasc Interv. 2015;8:e002230* |
| 95 | REWIND(45) | Prof Hertzel C Gestein | *Lancet 2019; 394:121-30* |
| 98 | SCOPE I(46) | Dr Thomas Pilgrim | *Lancet. 2019 Nov 2;394(10209):1619-1628* |
| 99 | SECURE-PCI(47) | Berwanger O | *JAMA. 2018 Apr 3;319(13):1331-1340* |
| 100 | SENIOR(48) | Olivier Varenne | *Lancet. 2018 Jan 6;391(10115):41-50* |
| 103 | SORT OUT II(49) | Dr. Anders M. | *J Am Coll Cardiol 2017;69:616–24* |
| 106 | SORT OUT IX(50) | Lisette Okkels Jensen | *Circulation 2020;141:2052-2063* |
| 107 | SPIRE-1 & SPIRE-2(51) | Dr. Ridker | *N Engl J Med. 2017;376(16):1527–39* |
| 109 | SUSTAIN(52) | Steven P. Marso | *N Engl J Med 2016;319(13):1331-1340* |
| 112 | TALENT(53) | Prof Patrick W Serruys | *Lancet. 2019 Mar 9;393(10175):987-997* |
| 114 | TECOS(54) | Dr. Holman | *N Engl J Med 2015;373:232-42* |
| 118 | TUXEDO(55) | U. Kaul, | *N Engl J Med 2015;373:1709-19* |
| 119 | TWENTE II(56) | Prof. Clemens von Birgelen | *JACC 2015* |
| 120 | ULTIMATE(57) | Dr. Shao-Liang Chen | *Am Coll Cardiol. 2018 Dec 18;72(24):3126-3137* |
| 125 | Yoga-Ca(58) | Dr Dorairaj Prabhakaran | *Journal of American College of Cardiology 2020:75; 1551-61* |
| 126 | ZEUS(59) | Marco Valgimigli | *J Am Coll Cardiol 2015;65:805–15* |
| 127 | CREDENCE(60) | Cannon C, | *Circulation. 2020;141(5):407-410* |
| 128 | PROMISE(61) | Dr Dougla | *New England Journal of medicine2015; 372: 1291-1300* |
| 130 | ACE(62) | Rury R. Holman, FRCP, FMedSci | *Lancet Diabetes and Endocrinology, 5(11), pp. 877-886* |
| 131 | ACS(7) | Martin Möckel | *European Heart Journal (2015) 36, 369–376* |
| 134 | GIK(63) | Dr. Jia Li, MD, PhD, and Dr. Feng Gao, MD, PhD and Dr. Feng Gao, MD, PhD | *J Am Heart Assoc. 2020;9:e012376.* |

*List of the 63 included Randomized Clinical Trials, acronym of the RCT, corresponding author and bibliographic reference.*

Table 3. Degree of association between each pair of events quantified by the Joint probability and the Correlation

| **RCT** | **N** | **Event 1** | | **Event 2** | | | **Association between both events** | | | | | |
| --- | --- | --- | --- | --- | --- | --- | --- | --- | --- | --- | --- | --- |
|  |  | **Label** | **n (%)** | **Label** | **n (%)** | **#** | | **Joint Probability** | **Correlation** | **Min.** | **Max.** |  |
|  |  |  |  |  |  |  | |  |  |  |  |  |
| ACCELERATE * | 12092 | CV death | 309 (2.6%) | AMI | 517 (4.3%) | 74 | | 0.61% | 0.157 | -0.034 | 0.766 |  |
| ACCELERATE | 12092 | CV death | 309 (2.6%) | Stroke | 192 (1.6%) | 35 | | 0.29% | 0.126 | -0.021 | 0.784 |  |
| ACCELERATE * | 12092 | CV death | 309 (2.6%) | Revascularization | 972 (8%) | 38 | | 0.31% | 0.025 | -0.048 | 0.548 |  |
| ACCELERATE | 12092 | CV death | 309 (2.6%) | Angina | 299 (2.5%) | 10 | | 0.08% | 0.008 | -0.026 | 0.983 |  |
| ACCELERATE | 12092 | AMI | 517 (4.3%) | Stroke | 192 (1.6%) | 25 | | 0.21% | 0.055 | -0.027 | 0.601 |  |
| ACCELERATE * | 12092 | AMI | 517 (4.3%) | Revascularization | 972 (8%) | 330 | | 2.73% | 0.434 | -0.062 | 0.715 |  |
| ACCELERATE | 12092 | AMI | 517 (4.3%) | Angina | 299 (2.5%) | 40 | | 0.33% | 0.07 | -0.034 | 0.753 |  |
| ACCELERATE | 12092 | Stroke | 192 (1.6%) | Revascularization | 972 (8%) | 26 | | 0.22% | 0.03 | -0.038 | 0.430 |  |
| ACCELERATE | 12092 | Stroke | 192 (1.6%) | Angina | 299 (2.5%) | 5 | | 0.04% | 0.00 | -0.020 | 0.798 |  |
| ACCELERATE | 12092 | Revascularization | 972 (8%) | Angina | 299 (2.5%) | 250 | | 2.07% | 0.44 | -0.047 | 0.539 |  |
| GIK | 930 | All-causes death | 20 (2.2%) | HF | 170 (18.3%) | 18 | | 1.94% | 0.28 | -0.070 | 0.313 |  |
| GIK * | 930 | All-causes death | 20 (2.2%) | AMI | 2 (0.2%) | 2 | | 0.22% | 0.31 | -0.007 | 0.313 |  |
| GIK | 930 | All-causes death | 20 (2.2%) | Low cardiac output syndrome | 36 (3.9%) | 15 | | 1.61% | 0.55 | -0.030 | 0.739 |  |
| GIK | 930 | HF | 170 (18.3%) | AMI | 2 (0.2%) | 2 | | 0.22% | 0.10 | -0.022 | 0.098 |  |
| GIK | 930 | HF | 170 (18.3%) | Low cardiac output syndrome | 36 (3.9%) | 36 | | 3.87% | 0.42 | -0.095 | 0.424 |  |
| GIK | 930 | AMI | 2 (0.2%) | Low cardiac output syndrome | 7 (0.8%) | 2 | | 0.22% | 0.53 | -0.004 | 0.533 |  |
| PROMISE * | 10003 | All-causes death | 149 (1.5%) | AMI | 70 (0.7%) | 3 | | 0.03% | 0.02 | -0.010 | 0.683 |  |
| PROMISE | 10003 | All-causes death | 149 (1.5%) | Angina | 102 (1%) | 0 | | 0.00% | -0.01 | -0.012 | 0.825 |  |
| PROMISE | 10003 | All-causes death | 149 (1.5%) | Other | 9 (0.1%) | 1 | | 0.01% | 0.02 | -0.004 | 0.244 |  |
| PROMISE | 10003 | AMI | 70 (0.7%) | Angina | 102 (1%) | 8 | | 0.08% | 0.09 | -0.009 | 0.827 |  |
| PROMISE | 10003 | AMI | 70 (0.7%) | Other | 9 (0.1%) | 0 | | 0.00% | 0.00 | -0.003 | 0.357 |  |
| PROMISE | 10003 | Angina | 102 (1%) | Other | 9 (0.1%) | 3 | | 0.03% | 0.10 | -0.003 | 0.296 |  |
| Yoga-Ca * | 3959 | All-causes death | 144 (3.6%) | AMI | 28 (0.7%) | 0 | | 0.00% | -0.02 | -0.016 | 0.434 |  |
| Yoga-Ca | 3959 | All-causes death | 144 (3.6%) | Stroke | 7 (0.2%) | 1 | | 0.03% | 0.02 | -0.008 | 0.217 |  |
| Yoga-Ca | 3959 | All-causes death | 144 (3.6%) | Other | 107 (2.7%) | 15 | | 0.38% | 0.09 | -0.032 | 0.858 |  |
| Yoga-Ca | 3959 | AMI | 28 (0.7%) | Stroke | 7 (0.2%) | 0 | | 0.00% | 0.00 | -0.004 | 0.499 |  |
| Yoga-Ca | 3959 | AMI | 28 (0.7%) | Other | 107 (2.7%) | 2 | | 0.05% | 0.02 | -0.014 | 0.506 |  |
| Yoga-Ca | 3959 | Stroke | 7 (0.2%) | Other | 107 (2.7%) | 0 | | 0.00% | -0.01 | -0.007 | 0.253 |  |
| MR.INFORM * | 918 | All-causes death | 6 (0.7%) | AMI | 19 (2.1%) | 1 | | 0.11% | 0.08 | -0.012 | 0.558 |  |
| MR.INFORM * | 918 | All-causes death | 6 (0.7%) | Revascularization | 10 (1.1%) | 0 | | 0.00% | -0.01 | -0.009 | 0.773 |  |
| MR.INFORM * | 918 | AMI | 19 (2.1%) | Revascularization | 10 (1.1%) | 3 | | 0.33% | 0.21 | -0.015 | 0.722 |  |

*N: Number of patients included in that RCT. n (%): Number and proportion of patients having each Event. #: Number of patients having both events. The joint proportion is defined as usually as the rate between the number of patients who had both events and the total number of patients on the RCT. The correlation not have a direct clinical interpretation. *: This pair of events has been considered for the study of the impact of the correlation.*

# Bibliography

1. Cyrne Carvalho H. Comment on “Randomised comparison of a bioresorbable everolimus‐eluting scaffold with a metallic everolimus‐eluting stent for ischaemic heart disease caused by de novo native coronary artery lesions: the 2‐year clinical outcomes of the ABSORB II trial.” Rev Port Cardiol. 2017;36(1):73–5.

2. Lincoff AM, Nicholls SJ, Riesmeyer JS, Barter PJ, Brewer HB, Fox KAA, et al. Evacetrapib and Cardiovascular Outcomes in High-Risk Vascular Disease. N Engl J Med. 2017;376(20):1933–42.

3. Thiele H, Wöhrle J, Hambrecht R, Rittger H, Birkemeyer R, Lauer B, et al. Intracoronary versus intravenous bolus abciximab during primary percutaneous coronary intervention in patients with acute ST-elevation myocardial infarction: A randomised trial. Lancet. 2012;379(9819):923–31.

4. Wang TY, Kaltenbach LA, Cannon CP, Fonarow GC, Choudhry NK, Henry TD, et al. Effect of Medication Co-payment Vouchers on P2Y12 Inhibitor Use and Major Adverse Cardiovascular Events among Patients with Myocardial Infarction: The ARTEMIS Randomized Clinical Trial. JAMA - J Am Med Assoc. 2019;321(1):44–55.

5. Wise RA, Chapman KR, Scirica BM, Bhatt DL, Daoud SZ, Zetterstrand S, et al. Effect of Aclidinium Bromide on Major Cardiovascular Events and Exacerbations in High-Risk Patients With Chronic Obstructive Pulmonary Disease: The ASCENT-COPD Randomized Clinical Trial. Jama. 2019;321(17):1693–701.

6. Montalescot G, van ’t Hof AW, Lapostolle F, Silvain J, Lassen JF, Bolognese L, et al. Prehospital Ticagrelor in ST-Segment Elevation Myocardial Infarction. N Engl J Med. 2014;371(11):1016–27.

7. Möckel M, Searle J, Hamm C, Slagman A, Blankenberg S, Huber K, et al. Early discharge using single cardiac troponin and copeptin testing in patients with suspected acute coronary syndrome (ACS): A randomized, controlled clinical process study. Eur Heart J. 2015;36(6):369–76.

8. Park S-J, Ahn J-M, Kim Y-H, Park D-W, Yun S-C, Lee J-Y, et al. Trial of Everolimus-Eluting Stents or Bypass Surgery for Coronary Disease. N Engl J Med. 2015;372(13):1204–12.

9. Ray KK, Nicholls SJ, Buhr KA, Ginsberg HN, Johansson JO, Kalantar-Zadeh K, et al. Effect of Apabetalone Added to Standard Therapy on Major Adverse Cardiovascular Events in Patients with Recent Acute Coronary Syndrome and Type 2 Diabetes: A Randomized Clinical Trial. JAMA - J Am Med Assoc. 2020;323(16):1565–73.

10. Iglesias JF, Muller O, Heg D, Roffi M, Kurz DJ, Moarof I, et al. Biodegradable polymer sirolimus-eluting stents versus durable polymer everolimus-eluting stents in patients with ST-segment elevation myocardial infarction (BIOSTEMI): a single-blind, prospective, randomised superiority trial. Lancet. 2019;394(10205):1243–53.

11. Dangas GD, Lefèvre T, Kupatt C, Tchetche D, Schäfer U, Dumonteil N, et al. Bivalirudin Versus Heparin Anticoagulation in Transcatheter Aortic Valve Replacement the Randomized BRAVO-3 Trial. J Am Coll Cardiol. 2015;66(25):2860–8.

12. Rosenstock J, Perkovic V, Johansen OE, Cooper ME, Kahn SE, Marx N, et al. Effect of Linagliptin vs Placebo on Major Cardiovascular Events in Adults with Type 2 Diabetes and High Cardiovascular and Renal Risk: The CARMELINA Randomized Clinical Trial. JAMA - J Am Med Assoc. 2019;321(1):69–79.

13. Linde JJ, Hove JD, Sørgaard M, Kelbæk H, Jensen GB, Kühl JT, et al. Long-Term Clinical Impact of Coronary CT Angiography in Patients with Recent Acute-Onset Chest Pain: The Randomized Controlled CATCH Trial. JACC Cardiovasc Imaging. 2015;8(12):1404–13.

14. Chang HJ, Lin FY, Gebow D, An HY, Andreini D, Bathina R, et al. Selective Referral Using CCTA Versus Direct Referral for Individuals Referred to Invasive Coronary Angiography for Suspected CAD: A Randomized, Controlled, Open-Label Trial. JACC Cardiovasc Imaging. 2019;12(7):1303–12.

15. Vlachojannis GJ, Smits PC, Hofma SH, Togni M, Vázquez N, Valdés M, et al. Biodegradable Polymer Biolimus-Eluting Stents Versus Durable Polymer Everolimus-Eluting Stents in Patients With Coronary Artery Disease: Final 5-Year Report From the COMPARE II Trial (Abluminal Biodegradable Polymer Biolimus-Eluting Stent Versus Durable P. JACC Cardiovasc Interv. 2017;10(12):1215–21.

16. Lee SW, Lee PH, Ahn JM, Park DW, Yun SC, Han S, et al. Randomized Trial Evaluating Percutaneous Coronary Intervention for the Treatment of Chronic Total Occlusion: The DECISION-CTO Trial. Circulation. 2019;139(14):1674–83.

17. Wiviott SD, Raz I, Bonaca MP, Mosenzon O, Kato ET, Cahn A, et al. Dapagliflozin and Cardiovascular Outcomes in Type 2 Diabetes. N Engl J Med. 2019;380(4):347–57.

18. Davies JE, Sen S, Dehbi H-M, Al-Lamee R, Petraco R, Nijjer SS, et al. Use of the Instantaneous Wave-free Ratio or Fractional Flow Reserve in PCI. N Engl J Med. 2017;376(19):1824–34.

19. de Winter RJ, Katagiri Y, Asano T, Milewski KP, Lurz P, Buszman P, et al. A sirolimus-eluting bioabsorbable polymer-coated stent (MiStent) versus an everolimus-eluting durable polymer stent (Xience) after percutaneous coronary intervention (DESSOLVE III): a randomised, single-blind, multicentre, non-inferiority, phase 3 trial. Lancet. 2018;391(10119):431–40.

20. Pfeffer MA, Claggett B, Diaz R, Dickstein K, Gerstein HC, Køber L V., et al. Lixisenatide in Patients with Type 2 Diabetes and Acute Coronary Syndrome. N Engl J Med. 2015;373(23):2247–57.

21. Wanner C, Inzucchi SE, Lachin JM, Fitchett D, von Eynatten M, Mattheus M, et al. Empagliflozin and Progression of Kidney Disease in Type 2 Diabetes. N Engl J Med. 2016;375(4):323–34.

22. Berger JS, Baumgartner I, Held P, Ph D, Katona BG, Pharm D, et al. Ticagrelor versus Clopidogrel in Peripheral Artery Disease. N Engl J Med. 2017;376(15):1487–9.

23. Holman RR, Bethel MA, Mentz RJ, Thompson VP, Lokhnygina Y, Buse JB, et al. Effects of Once-Weekly Exenatide on Cardiovascular Outcomes in Type 2 Diabetes. N Engl J Med. 2017;377(13):1228–39.

24. Van Nunen LX, Zimmermann FM, Tonino PAL, Barbato E, Baumbach A, Engstrøm T, et al. Fractional flow reserve versus angiography for guidance of PCI in patients with multivessel coronary artery disease (FAME): 5-year follow-up of a randomised controlled trial. Lancet. 2015;386(10006):1853–60.

25. Sabatine MS, Giugliano RP, Keech AC, Honarpour N, Wiviott SD, Murphy SA, et al. Evolocumab and Clinical Outcomes in Patients with Cardiovascular Disease. N Engl J Med. 2017;376(18):1713–22.

26. Hernandez AF, Green JB, Janmohamed S, D’Agostino RB, Granger CB, Jones NP, et al. Albiglutide and cardiovascular outcomes in patients with type 2 diabetes and cardiovascular disease (Harmony Outcomes): a double-blind, randomised placebo-controlled trial. Lancet. 2018;392(10157):1519–29.

27. Götberg M, Christiansen EH, Gudmundsdottir IJ, Sandhall L, Danielewicz M, Jakobsen L, et al. Instantaneous Wave-free Ratio versus Fractional Flow Reserve to Guide PCI. N Engl J Med. 2017;376(19):1813–23.

28. Cannon CP, Blazing MA, Giugliano RP, McCagg A, White JA, Theroux P, et al. Ezetimibe Added to Statin Therapy after Acute Coronary Syndromes. N Engl J Med. 2015;372(25):2387–97.

29. Didier R, Morice MC, Barragan P, Noryani AAL, Noor HA, Majwal T, et al. 6- Versus 24-Month Dual Antiplatelet Therapy After Implantation of Drug-Eluting Stents in Patients Nonresistant to Aspirin: Final Results of the ITALIC Trial (Is There a Life for DES After Discontinuation of Clopidogrel). JACC Cardiovasc Interv. 2017;10(12):1202–10.

30. Hong SJ, Kim BK, Shin DH, Nam CM, Kim JS, Ko YG, et al. Effect of intravascular ultrasound-guided vs angiography- guided everolimus-eluting stent implantation: The IVUS-XPL randomized clinical trial. JAMA - J Am Med Assoc. 2015;314(20):2155–63.

31. Nakamura M, Muramatsu T, Yokoi H, Okada H, Ochiai M, Suwa S, et al. Three-year follow-up outcomes of SES and PES in a randomized controlled study stratified by the presence of diabetes mellitus: J-DEsSERT trial. Int J Cardiol. 2016;208:4–12.

32. Mann JFE, Nauck MA, Nissen SE, Pocock S, Ph D, Zinman B, et al. Liraglutide and cardiovascular outcomes in type 2 diabetes. Drug Ther Bull. 2016;54(9):101.

33. Valgimigli M, Frigoli E, Leonardi S, Rothenbühler M, Gagnor A, Calabrò P, et al. Bivalirudin or Unfractionated Heparin in Acute Coronary Syndromes. N Engl J Med. 2015;373(11):997–1009.

34. Gantz I, Chen M, Suryawanshi S, Ntabadde C, Shah S, O’Neill EA, et al. A randomized, placebo-controlled study of the cardiovascular safety of the once-weekly DPP-4 inhibitor omarigliptin in patients with type 2 diabetes mellitus. Cardiovasc Diabetol. 2017;16(1):1–12.

35. Nagel E, Greenwood JP, McCann GP, Bettencourt N, Shah AM, Hussain ST, et al. Magnetic Resonance Perfusion or Fractional Flow Reserve in Coronary Disease. N Engl J Med. 2019;380(25):2418–28.

36. Holm NR, Mäkikallio T, Lindsay MM, Spence MS, Erglis A, Menown IBA, et al. Percutaneous coronary angioplasty versus coronary artery bypass grafting in the treatment of unprotected left main stenosis: updated 5-year outcomes from the randomised, non-inferiority NOBLE trial. Lancet. 2020;395(10219):191–9.

37. Schwartz GG, Steg PG, Szarek M, Bhatt DL, Bittner VA, Diaz R, et al. Alirocumab and Cardiovascular Outcomes after Acute Coronary Syndrome. N Engl J Med. 2018;379(22):2097–107.

38. Windecker S, Latib A, Kedhi E, Kirtane AJ, Kandzari DE, Mehran R, et al. Polymer-based or Polymer-free Stents in Patients at High Bleeding Risk. N Engl J Med. 2020;382(13):1208–18.

39. Notarangelo FM, Maglietta G, Bevilacqua P, Cereda M, Merlini PA, Villani GQ, et al. Pharmacogenomic Approach to Selecting Antiplatelet Therapy in Patients With Acute Coronary Syndromes: The PHARMCLO Trial. J Am Coll Cardiol. 2018;71(17):1869–77.

40. Husain M, Birkenfeld AL, Donsmark M, Dungan K, Eliaschewitz FG, Franco DR, et al. Oral Semaglutide and Cardiovascular Outcomes in Patients with Type 2 Diabetes. N Engl J Med. 2019;381(9):841–51.

41. Ahn JM, Roh JH, Kim YH, Park DW, Yun SC, Lee PH, et al. Randomized trial of stents versus bypass surgery for left main coronary artery disease: 5-year outcomes of the PRECOMBAT study. J Am Coll Cardiol. 2015;65(20):2198–206.

42. Bhatt DL, Steg PG, Miller M, Brinton EA, Jacobson TA, Ketchum SB, et al. Cardiovascular Risk Reduction with Icosapent Ethyl for Hypertriglyceridemia. N Engl J Med. 2019;380(1):11–22.

43. Zenati MA, Bhatt DL, Bakaeen FG, Stock EM, Biswas K, Gaziano JM, et al. Randomized Trial of Endoscopic or Open Vein-Graft Harvesting for Coronary-Artery Bypass. N Engl J Med. 2019;380(2):132–41.

44. Iqbal J, Serruys PW, Silber S, Kelbaek H, Richardt G, Morel MA, et al. Comparison of zotarolimus-and everolimus-eluting coronary stents: Final 5-year report of the RESOLUTE all-comers trial. Circ Cardiovasc Interv. 2015;8(6):1–8.

45. Gerstein HC, Colhoun HM, Dagenais GR, Diaz R, Lakshmanan M, Pais P, et al. Dulaglutide and cardiovascular outcomes in type 2 diabetes (REWIND): a double-blind, randomised placebo-controlled trial. Lancet. 2019;394(10193):121–30.

46. Lanz J, Kim WK, Walther T, Burgdorf C, Möllmann H, Linke A, et al. Safety and efficacy of a self-expanding versus a balloon-expandable bioprosthesis for transcatheter aortic valve replacement in patients with symptomatic severe aortic stenosis: a randomised non-inferiority trial. Lancet. 2019;394(10209):1619–28.

47. Berwanger O, Santucci EV, De Barros E Silva PGM, Jesuíno IDA, Damiani LP, Barbosa LM, et al. Effect of loading dose of atorvastatin prior to planned percutaneous coronary intervention on major adverse cardiovascular events in acute coronary syndrome the SECURE-PCI randomized clinical trial. JAMA - J Am Med Assoc. 2018;319(13):1331–40.

48. Varenne O, Cook S, Sideris G, Kedev S, Cuisset T, Carrié D, et al. Drug-eluting stents in elderly patients with coronary artery disease (SENIOR): a randomised single-blind trial. Lancet. 2018;391(10115):41–50.

49. Galløe AM, Kelbæk H, Thuesen L, Hansen HS, Ravkilde J, Hansen PR, et al. 10-Year Clinical Outcome After Randomization to Treatment by Sirolimus- or Paclitaxel-Eluting Coronary Stents. J Am Coll Cardiol. 2017;69(6):616–24.

50. Jensen LO, Ellert J, Veien KT, Ahlehoff O, Hansen KN, Aziz A, et al. Randomized Comparison of the Polymer-Free Biolimus-Coated BioFreedom Stent with the Ultrathin Strut Biodegradable Polymer Sirolimus-Eluting Orsiro Stent in an All-Comers Population Treated with Percutaneous Coronary Intervention: The SORT out IX Trial. Circulation. 2020;2052–63.

51. Ridker PM, Revkin J, Amarenco P, Brunell R, Curto M, Civeira F, et al. Cardiovascular Efficacy and Safety of Bococizumab in High-Risk Patients. N Engl J Med. 2017;376(16):1527–39.

52. Marso SP, Bain SC, Consoli A, Eliaschewitz FG, Jódar E, Leiter LA, et al. Semaglutide and Cardiovascular Outcomes in Patients with Type 2 Diabetes. N Engl J Med. 2016;375(19):1834–44.

53. Zaman A, de Winter RJ, Kogame N, Chang CC, Modolo R, Spitzer E, et al. Safety and efficacy of a sirolimus-eluting coronary stent with ultra-thin strut for treatment of atherosclerotic lesions (TALENT): a prospective multicentre randomised controlled trial. Lancet. 2019;393(10175):987–97.

54. Green JB, Bethel MA, Armstrong PW, Buse JB, Engel SS, Garg J, et al. Effect of Sitagliptin on Cardiovascular Outcomes in Type 2 Diabetes. N Engl J Med. 2015;373(3):232–42.

55. Bangalore S, Bhagwat A, Pinto B, Goel PK, Jagtap P, Sathe S, et al. Percutaneous coronary intervention in patients with insulin-treated and non-insulin-treated diabetes mellitus: Secondary analysis of the TUXEDO Trial. JAMA Cardiol. 2016;1(3):266–73.

56. Sen H, Lam MK, Löwik MM, Danse PW, Jessurun GAJ, Van Houwelingen KG, et al. Clinical Events and Patient-Reported Chest Pain in All-Comers Treated With Resolute Integrity and Promus Element Stents: 2-Year Follow-Up of the DUTCH PEERS (DUrable Polymer-Based STent CHallenge of Promus ElemEnt Versus ReSolute Integrity) Randomized Tri. JACC Cardiovasc Interv. 2015;8(7):889–99.

57. Zhang J, Gao X, Kan J, Ge Z, Han L, Lu S, et al. Intravascular Ultrasound Versus Angiography-Guided Drug-Eluting Stent Implantation: The ULTIMATE Trial. J Am Coll Cardiol. 2018;72(24):3126–37.

58. Prabhakaran D, Chandrasekaran AM, Singh K, Mohan B, Chattopadhyay K, Chadha DS, et al. Yoga-Based Cardiac Rehabilitation After Acute Myocardial Infarction: A Randomized Trial. J Am Coll Cardiol. 2020;75(13):1551–61.

59. Valgimigli M, Patialiakas A, Thury A, McFadden E, Colangelo S, Campo G, et al. Zotarolimus-eluting versus bare-metal stents in uncertain drug-eluting stent candidates. J Am Coll Cardiol. 2015;65(8):805–15.

60. Cannon CP, Perkovic V, Agarwal R, Baldassarre J, Bakris G, Charytan DM, et al. Evaluating the Effects of Canagliflozin on Cardiovascular and Renal Events in Patients with Type 2 Diabetes Mellitus and Chronic Kidney Disease According to Baseline HbA1c, including Those with HbA1c <7%: Results from the CREDENCE Trial. Circulation. 2020;407–10.

61. Douglas PS, Hoffmann U, Patel MR, Mark DB, Al-Khalidi HR, Cavanaugh B, et al. Outcomes of Anatomical versus Functional Testing for Coronary Artery Disease. N Engl J Med. 2015;372(14):1291–300.

62. Holman RR, Coleman RL, Chan JCN, Chiasson JL, Feng H, Ge J, et al. Effects of acarbose on cardiovascular and diabetes outcomes in patients with coronary heart disease and impaired glucose tolerance (ACE): a randomised, double-blind, placebo-controlled trial. Lancet Diabetes Endocrinol. 2017;5(11):877–86.

63. Zhao K, Zhang Y, Li J, Cui Q, Zhao R, Chen W, et al. Modified Glucose-Insulin-Potassium Regimen Provides Cardioprotection With Improved Tissue Perfusion in Patients Undergoing Cardiopulmonary Bypass Surgery. J Am Heart Assoc. 2020;9(6):e012376.
